# MSCs-Derived Exosomes: Cell-Secreted Nanovesicles with Regenerative Potential

**DOI:** 10.3389/fphar.2016.00231

**Published:** 2016-08-03

**Authors:** Ana Marote, Fábio G. Teixeira, Bárbara Mendes-Pinheiro, António J. Salgado

**Affiliations:** ^1^Life and Health Sciences Research Institute (ICVS), School of Health Sciences, University of Minho, BragaPortugal; ^2^ICVS/3B’s, PT Government Associate Laboratory, Braga/GuimarãesPortugal

**Keywords:** exosomes, mesenchymal stem cells, secretome, regeneration, cell-free therapy

## Abstract

Exosomes are membrane-enclosed nanovesicles (30–150 nm) that shuttle active cargoes between different cells. These tiny extracellular vesicles have been recently isolated from mesenchymal stem cells (MSCs) conditioned medium, a population of multipotent cells identified in several adult tissues. MSCs paracrine activity has been already shown to be the key mediator of their elicited regenerative effects. On the other hand, the individual contribution of MSCs-derived exosomes for these effects is only now being unraveled. The administration of MSCs-derived exosomes has been demonstrated to restore tissue function in multiple diseases/injury models and to induce beneficial *in vitro* effects, mainly mediated by exosomal-enclosed miRNAs. Additionally, the source and the culture conditions of MSCs have been shown to influence the regenerative responses induced by exosomes. Therefore, these studies reveal that MSCs-derived exosomes hold a great potential for cell-free therapies that are safer and easier to manipulate than cell-based products. Nevertheless, this is an emerging research field and hence, further studies are required to understand the full dimension of this complex intercellular communication system and how it can be optimized to take full advantage of its therapeutic effects. In this mini-review, we summarize the most significant new advances in the regenerative properties of MSCs-derived exosomes and discuss the molecular mechanisms underlying these effects.

## Introduction

Exosomes are the tiniest extracellular vesicles (EVs) involved in a complex intercellular communication system (Lamichhane et al., 2015). EVs are categorized as exosomes due to their endocytic origin, in contrast to microvesicles that are formed from budding of the plasma membrane and apoptotic bodies derived from fragments of dying cells (Merino et al., 2016). Along with other EVs, exosomes are released by several cell types, carrying active signals that are able to influence the activity of recipient cells. Since its discovery, this extracellular communication system has been hijacked in several ways. Firstly, exosome surface markers and molecular cargoes have been worthily challenged as potential diagnostic biomarkers (Muller, 2012; Dorayappan et al., 2016; Escudero et al., 2016). Secondly, due to their low immunogenicity, long half-life in circulation and ability to cross the brain-blood barrier (BBB), exosomes have also been explored as a nanodelivery system of therapeutic signals, such as small interfering RNAs (Sun et al., 2010; Alvarez-Erviti et al., 2011; Kalani et al., 2014). Finally, and surprisingly, unmodified exosomes secreted by stem or progenitor cells have also shown beneficial effects and have been put forward as mediators of the regenerative responses elicited, for instance, by mesenchymal stem cells (MSCs; Lai et al., 2010; Xin et al., 2012). Indeed, the effects of these cells on tissue regeneration and repair have been mainly attributed to their secreted factors (known as secretome) rather than its *trans*-differentiation capacity (Teixeira et al., 2013). Having this in mind, under the scope of the present review we will address the recent evidences of the regenerative potential of MSCs-derived exosomes into different pathological conditions.

## Exosomes As Cell-Secreted EVs

Exosomes and microvesicles are the most thoroughly studied classes of EVs. Both are membrane-enclosed vesicles, surrounded by a phospholipid layer and packed with cell-type specific combinations of proteins, lipids, coding, and non-coding RNAs (Lener et al., 2015). Once released into the extracellular milieu, EVs can be taken up by target cells of the microenvironment or carried to distant sites through biological fluids, from which they have already been isolated, including urine (Royo et al., 2016), breast milk (Zonneveld et al., 2014), blood (Baranyai et al., 2015), and cerebrospinal fluid (Street et al., 2012).

**FIGURE 1:**
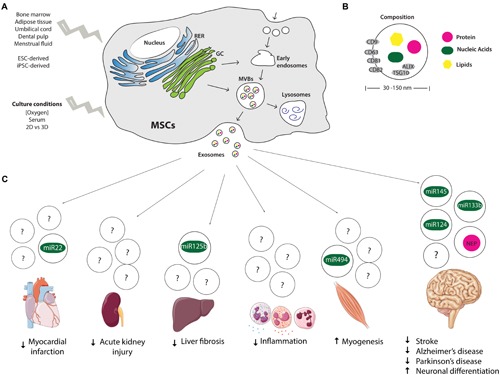
**Origin, composition, and therapeutic potential of MSCs-derived exosomes. (A)** Endocytic origin of MSCs-derived exosomes and influence of tissue from which MSCs are isolated and culture conditions on exosome secretion profile. **(B)** Size and composition of exosomes, depicting in gray the molecular markers of exosomes (CD9, 63, 81, and 82, ALIX and TSG10). **(C)** Therapeutic potential and suggested miRNAs and proteins mediating the regenerative effects of MSCs-derived exosomes. MSCs, mesenchymal stem cells; RER, rough endoplasmic reticulum; GC, Golgi complex; MVBs, multivesicular bodies; NEP, Neprilysin; ESC, embryonic stem cells; iPSCs, induced pluripotent stem cells.

In spite of their relative availability, the complexity of the extracellular environment and the existence of extracellular RNA in other non-EV carriers, such as protein complexes and lipoproteins, has encouraged the International Society for Extracellular Vesicles (ISEV; Lötvall et al., 2014) to establish minimal requirements for describing EVs, namely: (i) isolation from extracellular body fluids or conditioned cell culture medium, with minimal cell disruption; (ii) quantification of at least one protein from three different categories (transmembrane or lipid bound extracellular proteins, cytosolic proteins and intracellular proteins) in the EV preparation; (iii) characterization of single vesicles within the EV preparation using at least two different technologies, electron microscopy or atomic force microscopy (AFM) for imaging and nanoparticle-tracking analysis, dynamic light scattering, or resistive pulse sensing for size distribution measurements of EVs (Lötvall et al., 2014). EVs are further classified as exosomes or microvesicles based on their intracellular biogenesis pathway. Microvesicles are originated at the cell surface and released by direct outward budding of the plasma membrane, whereas exosomes are formed within multivesicular bodies (MVBs) at the endolysosomal pathway and secreted upon fusion of MVBs with the plasma membrane (**Figure 1A**; Kalani et al., 2014).

Exosomes comprise a homogenous population of nano-sized vesicles, as their diameter ranges from 30 to 150 nm (Lener et al., 2015; Willms et al., 2016). Additionally, exosomes are also a better characterized population of EVs. Exosomal proteins, RNAs and lipids described in published and unpublished studies have been cataloged in ExoCarta, a database that aims to identify specific molecular signatures of tissue/cell-type derived exosomes (Mathivanan et al., 2012). ALIX and TSG10 have been recognized as common protein markers for exosome identification, due to their participation in the endosomal sorting complex required for transport (ESCRT), as well as membrane proteins of the tetraspanin family, such as CD9, CD63, CD81, CD82 (**Figure 1B**; Wubbolts et al., 2003; Hurley and Odorizzi, 2012).

Conventional methods for isolation of exosomes include ultracentrifugation, density gradient separation, chromatography and immunoaffinity capture (IAC) techniques as well as commercial kits, such as polymer-based precipitation. Despite being all capable to isolate vesicles with exosomal diameter and markers, some authors have suggested IAC as the most effective method to isolate exosomes, based on the number of exosome-related proteins identified on the purified fraction (Tauro et al., 2012; Greening et al., 2015). However, there is an ongoing debate on the ability of these methods to fully separate exosomes from protein aggregates and other membranous vesicles. The distinction between microvesicles and exosomes, for instance, is challenging as a result of the heterogeneity of microvesicles diameter (50–1000 nm), as well as small amount of protein markers to clearly categorize EVs produced via budding from the cell membrane or produced via endosomal compartment. Therefore, the ISEV (Lötvall et al., 2014) has recommended cautious interpretation of some exosomal-mediated responses described so far. In fact, some studies focused on the evaluation of the individual contribution of exosomes and microvesicles, report unique effects of exosomes- and microvesicles- enriched fractions that were obtained through differential centrifugation steps (Jarmalaviciute et al., 2015; Lopez-Verrilli et al., 2016). Even within the same population of exosomes, Willms et al. (2016) have defined two distinct subpopulations of exosomes based on distinct particles sizes that enclose different protein and RNA compositions and, therefore, yield different effects on recipient cells. Taken together, these studies show that the improvement of current isolation methods and a better understanding of exosomal biology are of key importance to clearly determine the therapeutic effects of exosomes.

## MSCs and MSCs-Derived Exosomes

The regenerative potential of MSCs and particularly of their secretome has been extensively reviewed (Teixeira et al., 2013; Salgado et al., 2015; Konala et al., 2016). MSCs are a population of adult multipotent cells with the ability to self-renew and differentiate into mesenchymal lineages, namely osteoblasts, chondrocytes, and adipocytes (Pittenger et al., 1999). Even though originally isolated from bone marrow (BM-MSCs; Friedenstein et al., 1976), MSCs have been successfully identified and isolated from other adult tissues, such as adipose tissue (ASCs), dental pulp, placenta, amniotic fluid, umbilical cord blood, umbilical cord Wharton’s jelly (WJ-MSCs) and its perivascular region (HUCPVCs), and even the brain (Teixeira et al., 2013). MSCs differentiation potential has been explored in cell replacement strategies aiming to restore compromised adult mesenchymal tissues, although some authors have also reported its differentiation into ectodermal lineages (Donega et al., 2014; Takeda and Xu, 2015; Bagher et al., 2016). Nonetheless, the paradigm of MSCs-mediated regeneration has been shifting toward a secretome-based paracrine activity, rather than its cellular engraftment and differentiation. Indeed, it has been widely accepted that MSCs secrete bioactive factors with strong immunomodulatory activities, which are also able to inhibit fibrosis and apoptosis, enhance angiogenesis and promote neuronal survival and differentiation (Caplan and Dennis, 2006; Teixeira et al., 2015). As a result, the use of MSCs conditioned medium (CM) has been put forward as a cell-free strategy with promising therapeutic effects, including reduction of myocardial infarct size (Timmers et al., 2007), trophic support to injured liver (van Poll et al., 2008) and protective effects on acute kidney injury (Zarjou et al., 2011).

Initial studies from Timmers et al. (2007) on ischemia and reperfusion injury, demonstrated that only the fraction of the CM containing products > 1000 kDa provided cardioprotection. At that time, the authors suggested the existence of a complex of proteins and lipids (with a predicted diameter of 50–100 nm) mediating MSCs paracrine activity. Three years later, the same group (Lai et al., 2010) isolated and characterized exosomes from human ESC-derived MSCs CM, for the first time. These spherical structures were readily visualized at the CM by electron microscopy, and further precipitated by ultracentrifugation. The presence of exosomes was also evidenced by the enrichment in plasma membrane phospholipids, such as cholesterol, sphingomyelin and phosphatidylcholine, as well as coprecipitation of exosome-associated proteins, including CD81, CD9, and ALIX. Thenceforward, a novel perspective of intercellular mediation of tissue repair was highlighted.

Enclosed RNA molecules have been described as the main messengers of the responses induced by exosomes. Indeed, coding mRNA molecules packed within exosomes are functionally translated at recipient cells (Valadi et al., 2007). Notwithstanding, small non-coding RNAs (20–30 nucleotides), and particularly microRNAs (miRNA), have been described as major mediators of cell–cell communication as they are able to regulate gene expression via miRNA degradation and repression of translation (Ha and Kim, 2014). A small RNA profiling of BM-MSCs and ASCs exosomes, recently performed by Baglio et al. (2015), revealed that, along with miRNA, MSCs exosomes are highly enriched in transfer RNA (tRNA) species that can function as miRNAs. Furthermore, while cellular small RNA expression profile is not donor- or tissue-specific, exosomal small RNA content seems to be greatly influenced by cell differentiation status, i.e., donor variability, and cell type (Baglio et al., 2015). The authors suggest that exosomal-enclosed small RNAs released by MSCs may distinctly control the microenvironment in their resident niches through a balance between proliferation and differentiation (Baglio et al., 2015). This study shed light on possible regulatory mechanisms of MSCs paracrine activity responsible for tissue-specific MSCs CM regenerative properties that were previously described by our laboratory (Ribeiro et al., 2012). Recently, tissue-specific responses were also described for exosomes isolated from different sources. For instance, ASCs-derived exosomes seem to be more effective in degrading Aβ in an *in vitro* model of Alzheimer’s disease (AD), when compared to BM-MSCs-derived exosomes (Katsuda et al., 2013), whereas neurite outgrowth response appears to be enhanced by exosomes released by menstrual fluid derived MSCs, when compared to umbilical cord, chorion and bone marrow (Lopez-Verrilli et al., 2016). These studies unveil the need to further distinguish the functional properties of exosomes released by MSCs isolated from different adult tissues to better understand their individual regenerative potential. The influence of gender on MSCs exosomal secretion profile should be also taken into account in future studies.

## MSCs-Derived Exosomes Applied to Tissue Regeneration

Since the report of MSCs-derived exosomes, a growing amount of studies have explored their regenerative potential using different *in vitro* and *in vivo* models (**Figure 1C**). In the present report we will focus on those that, in our opinion, are the most relevant.

### Cardiovascular System

MSCs-derived exosomes were firstly suggested as cardioprotective agents by Kleijn’s group (Lai et al., 2010). Subsequently, other studies have addressed the proangiogenic effects of MSCs exosomes as a mechanism of tissue repair upon ischemia. Bian et al. (2014) firstly showed that MSCs secrete a large quantity of exosomes under hypoxia and serum deprivation. In a similar ischemic conditioning, MSCs were shown to secrete exosomes enriched with miR-22, which led to the reduction of apoptosis and cardiac fibrosis through a direct targeting of methyl CpG binding protein 2 (Mecp2; Feng et al., 2014). In line with this, a recent analysis of the proteomic profile of MSCs-derived exosomes cultured under these conditions revealed a robust profile of angiogenic signaling proteins, including NFkB interacting proteins, which mediate tubule formation upon low to medium doses (1–10 μg/mL) treatment of MSCs-derived exosomes (Anderson et al., 2016).

Importantly, several authors have demonstrated that internalization of MSCs-derived exosomes by endothelial cells stimulate proliferation, migration and tube formation (*in vitro*) and promote angiogenesis when injected intramyocardially in acute myocardial infarction rat models (Bian et al., 2014; Teng et al., 2015). Hu et al. (2015) have also evidenced that intramuscular injection of exosomes secreted by human induced pluripotent stem cells-derived MSCs (iPSCs-derived MSCs) promote endothelial cell migration, proliferation, and tube formation through the activation of angiogenesis-related molecule expression (VEGF, TGFB1, and Angiogenin), leading to an increase of microvessel density and blood perfusion in a mouse hind-limb ischemic model.

In a follow-up study, Arslan et al. (2013) have shown that a single intravenous administration of exosomes was effective in enhancing cardiac function and geometry after myocardial infarction due to bioenergetics reestablishment (increased ATP production), oxidative stress reduction and pro-survival signaling activation (enhanced PI3K/Akt signaling). A similar outcome was presented by Wang et al. (2015) in polymicrobial sepsis.

Asahara’s group (Zhang et al., 2016b) have used the proliferative and angiogenic potential of MSCs exosomes to condition cardiac stem cells (CSC) *in vitro*, which when transplanted into a rat model of myocardial infarction were able to preserve cardiac function.

### Kidney

Acute kidney injury has also been effectively treated with MSCs exosomes. *In vitro*, treated renal tubular epithelial cells exhibited increased activation of extracellular-signal-regulated kinase (ERK)1/2 pathway and thus increased cell proliferation (Zhou et al., 2013). *In vivo*, after direct injection into injured kidneys (cisplatin-induced acute kidney injury rat model), hucMSCs-Ex were shown to be incorporated into the tubular epithelial cells and to promote functional and morphologic recovery by reducing oxidative stress and suppressing apoptosis (Zhou et al., 2013). Recently, Jiang et al. (2016), characterized urine-derived stem cells as multipotent MSCs (USCs) and a source of exosomes, and demonstrated that when intravenously injected they were able to reduce kidney deficits in type I diabetic rats.

### Liver

HucMSCs-Ex have also been explored as a treatment to fibrotic liver disease, which can be induced by viral hepatitis, alcohol, drugs, metabolic diseases, and autoimmune attack of hepatic cells (Li et al., 2013). Li et al. (2013), have demonstrated that after liver fibrosis [induced by carbon tetrachloride (CCl4)], the delivery of exosomes into the liver reduced fibrosis through the inactivation of the transforming growth factor (TGF)-β 1/Smad signaling pathway, as well as through the inhibition of epithelial-to-mesenchymal transition of hepatocytes. Using the same mouse model, Hyun et al. (2015) demonstrated that chorionic plate-derived MSCs retained miR-125b in exosomes which suppressed the Hedgehog activation and consequently liver fibrosis. In addition to this, the hepatic regenerative potential of hESC-derived MSCs exosomes was also evidenced using two *in vitro* models of liver injury [acetaminophen (APAP)- and (2) hydrogen peroxide (H_2_O_2_)], in which treatment resulted in increased anti-apoptotic and proliferative responses (Tan et al., 2014).

### Immune System

In accordance to well-known MSCs immunomodulatory effects, MSCs exosomes have also been described as immunological active agents as they induce high levels of anti-inflammatory cytokines, in contrast to decreased levels of pro-inflammatory molecules, providing a rationale for the use of exosomes for the treatment of immune disorders (Zhang et al., 2014). In addition, MSCs exosomes can also inhibit macrophage activation by suppressing Toll-like receptor signaling, providing a physiologically relevant context for the innate immunomodulatory activity of MSCs (Phinney et al., 2015). In a mouse model of hypoxic pulmonary hypertension, Lee et al. (2012) demonstrated that the intravenous delivery of exosomes derived from MSCs CM suppress hypoxic inflammation by inhibition of pro-proliferative pathways, such as STAT3 phosphorylation.

### Musculoskeletal System

In the context of bone regeneration, Narayanan et al. (2016) demonstrated the potential of exosomes released by MSCs cultured under osteogenic conditions. In a 2D and in a 3D (type I collagen hydrogels) culture these exosomes triggered osteogenic differentiation of undifferentiated MSCs. *In vivo*, when implanted subcutaneously in a grade collagen membrane, “osteogenic” exosomes promoted calcium deposition and calcium phosphate nucleation. In addition, Nakamura et al. (2015) have also suggested that MSCs-derived exosomes are able to promote skeletal muscle regeneration by enhancing myogenesis and angiogenesis and have considered exosome-enclosed miR-494 an important mediator of this response.

### Nervous System

MSCs-secretome has shown therapeutic benefits for the treatment of neurological and neurodegenerative diseases. Accordingly, Xin et al. (2012, 2013b) have demonstrated that miR-133b, increased in MSCs exposed to ischemic cerebral extracts, is transferred to neurons and astrocytes via exosomes and promotes neurite outgrowth and functional recovery in a middle cerebral artery occlusion model. In another study, these authors (Xin et al., 2013a) demonstrated that after stroke, the intravenous administration of cell-free MSCs-generated exosomes also enhances neurite remodeling, neurogenesis and angiogenesis and therefore, improves functional recovery. Similar encouraging outcomes were obtained in a traumatic brain injury model, in which intravenous administration of MSCs-generated exosomes enhanced angiogenesis and neurogenesis, reduced inflammation and improved spatial learning and sensimotor function (Zhang et al., 2015; Kim et al., 2016).

Katsuda et al. (2013) have provided an important role for ASCs-derived exosomes in the context of AD, as these express high levels of Neprilysin (NEP), the most important Aβ-degrading enzyme in the brain. NEP-loaded exosomes were shown to be efficiently transferred to neuroblastoma cells and led to the decrease of both extracellular and intracellular Aβ levels. In another *in vitro* neurodegeneration model, exosomes released by dental pulp-derived MSCs on a 3D culture, rescued dopaminergic neurons from 6-OHDA induced apoptosis, thereby providing a possible treatment of Parkinson’s disease (Jarmalaviciute et al., 2015).

Other studies have focused on non-diseased *in vitro* models to demonstrate MSCs-derived exosomes potential for neuronal differentiation. For instance, Lee et al. (2014) reported that BM-MSCs deliver miR-124 and miR-145 to human neural progenitor cells (NPCs) and astrocytes via contact independent and exosome-dependent processes, which alter gene expression in the recipient neural cells. Indeed, exosomal-mediated axonal growth seems to depend on argonaut 2 protein, a miRNA machinery protein, whereas internalization of BM-MSCs exosomes in cell bodies and axons has been suggested to occur via SNARE complex (Zhang et al., 2016a). Lopez-Verrilli et al. (2016) have recently unraveled the potential of menstrual MSCs (MenSCs) exosomal-enriched fraction as therapeutic approach in neurodegenerative pathologies. These authors demonstrated that MenSC exosomes induced neurite growth in cortical neurons and had a similar effect to BM-SC exosomes on neurite outgrowth of dorsal root ganglia neurons.

## Conclusion and Future Perspectives

MSCs-derived exosomes have emerged, in the last six years, as an attractive and safe approach for regenerative medicine applications. Of note, the intravenous administration of these nanovesicles has not elicited adverse effects. In addition, MSCs-derived exosomes regenerative effects reinforce the paradigm of secretome-based paracrine action of MSCs and support future perspectives for the development of cell-free therapies that avoid the safety concerns associated to stem cell transplantation and are less technically difficult to prepare and deliver. Nevertheless, this is a still developing research area that needs to be optimized to take full advantage of MSCs-derived exosomes regenerative potential. For instance, the choice of MSCs source and their culture conditions must be explored, as these have been shown to impact the functional properties of the exosomes.

## Author Contributions

AM drafted the manuscript. FT helped to draft the manuscript and revised it critically. BM-P collected all scientific literature for review. AS conceived the analysis, participated in its design and coordination, helped to draft the manuscript and gave the final approval of the version to be published. All authors read and approved the final manuscript.

## Conflict of Interest Statement

The authors declare that the research was conducted in the absence of any commercial or financial relationships that could be construed as a potential conflict of interest.
